# The Hospital World

**Published:** 1911-01-07

**Authors:** 


### The Hospital World.

### Personalia.

Mr. George Macculloch Miller, President of the Hospital Saturday and Sunday Association of New York, since its inception thirty years ago, has recently resigned, and has been made honorary president. Mr. Robert Olyphant has been elected to fill the office thus made vacant.

Mr. Charles Cutting, the Secretary of the Royal Hospital for Incurables, Putney Heath, has been presented with his portrait in oils. The picture is the work of one of the patients of the hospital, who is an artist of ability.

Mr. Fred Smith, who has been cashier and assistant-secretary of the Bradford Royal Infirmary for about seven years, has been appointed house governor and secretary of the Warneford Hospital, Leamington Spa. There were sixty applications for the post, and the short list contained five names, four of these being hospital-trained men. We congratulate Mr. Smith on his new appointment, and the Warneford Hospital on securing so able and energetic a superintendent.

Three years ago, in 1907, Mr. John Birkmyre, of Renfrew, N.B., founded the Broadstone Jubilee Hospital, Port Glasgow. His death, which has been noted, apart from any other reason, on account of the large sum he left, will come, in spite of his advanced age, as a blow to the friends of the hospital. His practical interest in the institution which he founded can find no simpler proof than in the proviso which accompanied his bequest to it in his will. The £5,000 which he left to the trustees of the hospital is to be applied to the endowment fund, which shows his appreciation of the need of invested funds by the voluntary hospitals. There are twenty-three beds at the Broadstone Hospital, of which it may be said that almost a quarter have been endowed by the munificence of its founder.

The Bridgwater Hospital has not let 1910 pass without recording the great personalities in its history in visible form. First of all, the *primus inter pares*, is the bust of Dr. Jonathan Toogood, the pious founder, who started the institution in 1813. His relations have made the presentation, which, though long delayed, does, perhaps, him and them the greater honour. Two other personalities have been commemorated, namely Dr. F. J. C. Parsons and Dr. W. L. Winterbotham, whose work for the hospital has been recorded on brass tablets, and we hope that Mr. H. W. Pollard, to whose suggestion the setting up of these tablets was due, will be supported in his desire that the hospital's walls should record something at least of the men who worked within them.

### Elections and Appointments.

At the annual meeting of the Norwich Hospitals and Medical Charities Saturday and Sunday Fund, the following ladies and gentlemen were re-elected governors of the institution : County churches and chapels : the Ven. Archdeacon Collyer. Schools : Mr. J. Thorne. House-to-house collection : Mrs. J. R. Harvey. Workshops and factories : Mr. S. J. Ransome, Mr. John Blake, Mr. J. Gibson, Mr. W. B. Greenfield (G.E.R. Locomotive Department, Thorpe), Mr. A. Gowing, Mr. J. M. Lanham, Mr. A. T. Borrett, Mr. J. Canham, and Mr. H. W. Seaman.

The following have been elected vice-presidents of the Coventry and Warwickshire Hospital : The Earl of Denbigh, Lord Leigh, Mr. F. A. Newdigate-Newdegate, M.P., Mr. C. J. Murray, Mr. J. S. Dugdale, K.C., Dr. E. Lynes, Mr. D. M. Mason, M.P., Mr. A. E. W. Mason, and Mr. J. Kenneth Foster. Mr. Sidney T. Pierson has been re-appointed as auditor, while Canon Beaumont, Canon Evans, Canon Wood, Mr. B. Riley, Mr. E. Rotherham, Mr. A. Seymour, Mr. Edgar Turrall, Mr. W. J. Wormell, and also Mr. A. E. Bennett, Mr. E. M. C. Instone, and Mr. J. D. Siddeley were elected to act upon the General Committee for the ensuing year.

